# Swine-to-Ferret Transmission of Antigenically Drifted Contemporary Swine H3N2 Influenza A Virus Is an Indicator of Zoonotic Risk to Humans

**DOI:** 10.3390/v15020331

**Published:** 2023-01-24

**Authors:** Carine K. Souza, J. Brian Kimble, Tavis K. Anderson, Zebulun W. Arendsee, David E. Hufnagel, Katharine M. Young, Phillip C. Gauger, Nicola S. Lewis, C. Todd Davis, Sharmi Thor, Amy L. Vincent Baker

**Affiliations:** 1 Virus and Prion Research Unit, National Animal Disease Center, United States Department of Agriculture-Agricultural Research Service, Ames, IA 50010, USA; 2 Department of Veterinary Diagnostic and Production Animal Medicine, College of Veterinary Medicine, Iowa State University, Ames, IA 50011, USA; 3 Department of Pathology and Population Sciences, Royal Veterinary College, University of London, Hertfordshire, London NW1 0TU, UK; 4 Influenza Division, National Center for Immunization and Respiratory Diseases, Centers for Disease Control and Prevention, Atlanta, GA 30333, USA

**Keywords:** influenza A virus, H3N2, swine, pandemic preparedness, zoonosis, variant, antigenic drift

## Abstract

Human-to-swine transmission of influenza A (H3N2) virus occurs repeatedly and plays a critical role in swine influenza A virus (IAV) evolution and diversity. Human seasonal H3 IAVs were introduced from human-to-swine in the 1990s in the United States and classified as 1990.1 and 1990.4 lineages; the 1990.4 lineage diversified into 1990.4.A–F clades. Additional introductions occurred in the 2010s, establishing the 2010.1 and 2010.2 lineages. Human zoonotic cases with swine IAV, known as variant viruses, have occurred from the 1990.4 and 2010.1 lineages, highlighting a public health concern. If a variant virus is antigenically drifted from current human seasonal vaccine (HuVac) strains, it may be chosen as a candidate virus vaccine (CVV) for pandemic preparedness purposes. We assessed the zoonotic risk of US swine H3N2 strains by performing phylogenetic analyses of recent swine H3 strains to identify the major contemporary circulating genetic clades. Representatives were tested in hemagglutination inhibition assays with ferret post-infection antisera raised against existing CVVs or HuVac viruses. The 1990.1, 1990.4.A, and 1990.4.B.2 clade viruses displayed significant loss in cross-reactivity to CVV and HuVac antisera, and interspecies transmission potential was subsequently investigated in a pig-to-ferret transmission study. Strains from the three lineages were transmitted from pigs to ferrets via respiratory droplets, but there were differential shedding profiles. These data suggest that existing CVVs may offer limited protection against swine H3N2 infection, and that contemporary 1990.4.A viruses represent a specific concern given their widespread circulation among swine in the United States and association with multiple zoonotic cases.

## 1. Introduction

The H3N2 subtype of influenza A virus (IAV) has caused influenza morbidity and mortality in humans globally, with more severe annual epidemics than H1N1 or influenza B virus since the 1968 pandemic [1]. From 1968 to the present, human seasonal H3N2 strains have been introduced into pig populations, establishing endemic lineages that cause an important respiratory disease in pigs, impacting the swine industry, and posing a zoonotic health concern for humans. Pigs may serve as an intermediate host of IAV due to the expression of both α2,6 and α2,3 linked sialic acids on receptors, which also serve as IAV receptors in humans and birds, respectively [2]. Pigs may be infected by avian or human-derived IAV, although host barriers appear to limit the frequency of these interspecies events, particularly from birds to pigs [2]. When there is interspecies transmission, there may be rapid evolution within the swine host due to reassortment events and the error-prone replication of the virus. The evolution of human and avian IAV within the swine host has led to the emergence of novel strains with pandemic potential [3,4]. A notable example of the two-way transmission of IAV between humans and swine populations worldwide was the emergence of the 2009 pandemic H1N1 (H1N1pdm09) of swine-origin and subsequent transmission from humans to swine, resulting in reassortment with endemic swine IAV, including North American swine H3N2 viruses [5,6].

In the 1990s, the introduction of a human seasonal H3N2 virus into swine and the subsequent reassortment into viruses containing a triple reassortant internal gene cassette (TRIG) [4,7] dramatically increased endemic swine IAV diversity in North America [6,8,9]. This virus lineage had gene segments derived from classical swine lineage H1N1, human seasonal H3N2 viruses, and North American avian IAV [6,8,9]. The H3 genes from human seasonal introductions maintained in swine are phylogenetically distinct and referred to as the 1990.1 and 1990.4 clades using global nomenclature based on decade of introduction [10]. The 1990.4 lineage diversified into six phylogenetic clades (1990.4.A–F) [11,12]. More recent human-to-swine H3N2 transmission events in the 2010 decade led to the 2010.1 and 2010.2 lineages in U.S. swine from incursions occurring in 2010–11 and in 2016–17, respectively [13,14,15]. 

Human infections with IAV of swine origin, termed variant viruses to differentiate them from human seasonal IAV, are sporadically detected in the U.S. To date, a total of 434 H3N2 variant cases have been detected, with most cases involving direct or indirect contact with infected swine at interfaces such as agricultural fairs and livestock shows [13]. Most of these cases occurred in 2011–2012 (*n* = 306) and were caused by the 1990.4.A swine lineage, but there have been variant cases detected from other 1990.4 genetic clades along with the 1990.1 and 2010.1 lineages [10]. Although human-to-human transmission of variant IAV strains is rare, the zoonotic potential and/or pandemic risk of swine-origin IAV should not be underestimated, as exemplified by the H1N1pdm09 pandemic [3]. Variant IAV cases in the U.S. are monitored and reported by the Centers for Disease Control and Prevention (CDC), which then reports novel influenza viruses to the World Health Organization (WHO). Animal influenza activity, including variant detections, is reviewed twice per year during the WHO consultation meetings on the composition of IAV vaccines. If these variant viruses are genetically and antigenically distinct from current human seasonal vaccines and existing pre-pandemic candidate vaccine viruses (CVVs), a representative strain from the clade may be considered for the development of a new CVV [16]. Over the past decade, several avian and swine IAV strains from human zoonotic infections have been selected as CVVs and are available within the WHO Global Influenza Surveillance and Response System (GISRS). This initiative helps the international community prepare for the public health risks of animal influenza viruses with a potential global impact [17]. 

We previously identified swine H3N2 strains against which humans are likely to lack population immunity or are not protected by a current human seasonal vaccine, or CVV [18]. Adult human sera revealed limited immunity against the 1990.1, 1990.4.A, and 1990.4.B swine lineages, especially in individuals born after 1970 [18]. To further understand the zoonotic potential of swine IAV, we analyzed contemporary swine H3 HA genes collected in 2020 in the U.S. and selected representatives from each detected clade. Then, we used serological methods to test ferret antisera raised against human vaccines or related variant CVV strains to identify swine strains with limited cross-reactivity. Lastly, three representative H3N2 strains that showed a substantial reduction in cross-reactivity to a relevant CVV or a human seasonal vaccine were tested for zoonotic potential in a pig-to-ferret interspecies transmission model.

## 2. Material and Methods

### 2.1. Genetic Analysis and Strain Selection 

At the biannual WHO information meeting on the composition of influenza virus vaccines, animal influenza activity data for 6-month periods are presented and compared against human IAV vaccine components and pre-pandemic CVVs. All swine H3 HA sequences collected between 1 January 2020, and 30 June 2020 were downloaded from GISAID [19]. Sequences were compiled with human seasonal vaccine and CVV strains and aligned using MAFFT v7.453 [20]. We inferred a maximum-likelihood phylogeny for the HA nucleotide alignment using IQ-TREE v2 implementing automatic model selection [21,22] with subsequent tree visualization and annotation in Smot v.1.0.0 [23]. The HA genes and associated available whole genome data were classified to a genetic clade or evolutionary lineage using the octoFLU pipeline [24], and a consensus HA1 for each identified clade was generated from the translated amino acid sequence data using flutile (https://github.com/flu-crew/flutile, accessed on 5 December 2022). We identified lineages circulating in the U.S. and selected a representative strain for each genetic clade by generating a pairwise distance matrix and choosing the best match between the HA1 clade consensus sequence and a virus isolate available in the USDA IAV swine virus repository. We excluded the 1970.1, 2000.3, and Other-Human-1990 lineages as they were not detected in the U.S. We did not identify field isolates for additional assessment from the 2010.2 lineage because it was less frequently detected and retained cross-reactivity with human seasonal vaccine anti-sera [14] or the 2010.1 lineage because it includes a within-clade CVV that was previously characterized in our swine-to-ferret model [25], and human sera contained cross-reactive antibodies to this clade [18]. Selected viruses were tested by hemagglutinin inhibition (HI) assay (using guinea pig red blood cells) against ferret antisera raised against human seasonal vaccine viruses and CVVs. For swine-to-ferret transmission studies, we expanded the selection criteria to ensure the identified strain included a representative neuraminidase (NA) and internal gene constellation through identifying the predominant evolutionary lineages and genetic clades paired to the HA genes using octoFLUshow [24]. We identified the amino acid differences between the characterized strains, clade consensus HA1 sequences, and within-clade CVVs, or human seasonal vaccine strains, using flutile (https://github.com/flu-crew/flutile, accessed on 5 December 2022).

### 2.2. Viruses and Ferret Antisera 

Swine H3N2 isolates were selected from clades 1990.1 (A/swine/Missouri/A02257614/2018), 1990.4.A (A/swine/North Carolina/A02245294/2019) and 1990.4.B.2 (A/swine/Illinois/A02479007/2020) and provided by the National Veterinary Services Laboratories (NVSL) through the U.S. Department of Agriculture (USDA) IAV swine surveillance system in conjunction with the USDA-National Animal Health Laboratory Network (NAHLN). The virus isolates A/Minnesota/11/2010 × 203 CVV, IDCDC-RG55C (A/Ohio/28/2016-like) CVV, A/Indiana/27/2018 variant, and A/Iowa/60/2018, a human H3N2 seasonal vaccine strain (the A (H3N2) component of non-egg-based vaccines used in the 2020 Southern Hemisphere vaccine), along with ferret antisera produced against these strains, were provided from the CDC, Atlanta, Georgia, U.S.A. The viruses was propagated on Madin-Darby canine kidney (MDCK) cells grown in Opti-MEM (Life Technologies, Waltham, MA, USA). Virus growth media contained antibiotics/antimycotics and 1μg/mL of tosylsulfonyl phenylalanyl chloromethyl ketone (TPCK)-trypsin (Worthington Biochemical Corp., Lakewood, NJ, USA).

### 2.3. Hemagglutination Inhibition

Prior to hemagglutination inhibition (HI) assays, ferret antisera were heat inactivated at 56 °C for 30 min then treated with a 20% Kaolin suspension (Sigma-Aldrich, St. Louis, MO, USA), followed by an adsorption with 0.75% guinea pig red blood cells to remove nonspecific hemagglutination inhibitors, as previously described [26]. In order to address coverage of contemporary U.S. swine H3N2 viruses by the nearest CVV or HuVac, HI assays were performed using ferret antisera in the presence of 20 nM of oseltamivir carboxylate [10]. Pig antisera were treated with receptor-destroying enzyme (RDE) (Denka Seiken Co., LTD., Tokyo, Japan), heat inactivated at 56 °C for 30 min, and adsorbed with 50% turkey red blood cells for nonspecific hemagglutination inhibitors.

### 2.4. Swine-to-Ferret Transmission Study Design

A total of 20 three-week-old pigs obtained from a herd free of IAV and porcine reproductive and respiratory syndrome virus were housed in a biosafety level 2 containment facility in compliance with an approved USDA-ARS NADC animal care and use protocol. Upon arrival, pigs were treated prophylactically with ceftiofur (Zoetis, Florham Park, NJ, USA), according to the label directions, to reduce potential respiratory bacterial pathogens. Sixteen 4–6-month-old male and female ferrets were obtained from an influenza-free, high health source for use as transmission contacts. Upon arrival, all pigs and ferrets were screened for antibody against influenza A nucleoprotein (NP) by a commercial enzyme-linked immunosorbent assay (ELISA) (MultiS ELISA; Idexx, Westbrook, ME, USA) to ensure the absence of preexisting immunity from prior exposure or passively acquired maternal antibody. Pigs were randomly assigned into four groups of five (three experimental groups, one naïve control group) and placed into separate containment rooms. 

Pigs in experimental groups were challenged with 2 mL intranasally of 1 × 10^6^ 50% tissue culture infectious dose (TCID_50_)/mL [27] of each strain diluted in phosphate-buffered saline (PBS). At 2 days post infection (dpi), four ferrets per group were housed individually in open-front isolators were placed approximately 4 feet from the pig deck, as previously described [25]. All animals received a subcutaneous radio frequency microchip (pigs: Deston Fearing, Dallas, TX; Ferrets: Biomedic Data Systems Inc., Seaford, DE, USA) for identification and body temperature monitoring purposes. The body temperatures of contact ferrets were monitored from −2 to 12 dpc. A febrile response was considered when a ferret displayed a temperature greater than 2 standard deviations above the mean of ferret temperatures before exposure (>39.5 °C). Ferrets were provided routine care and handled before pigs, with a change in outer gloves and surface decontamination of gowns and equipment with 70% ethanol between individual ferrets.

Nasal swab samples (FLOQSwabs; Copan Diagnostics, Murrieta, CA, USA) were collected from pigs at 0, 1, 3, and 5 dpi, and then three pigs from each group were humanely euthanized and necropsied at 5 dpi to evaluate lung lesions, as previously described [28]. The remaining two pigs in each group were swabbed at 7 and 9 dpi, then humanely euthanized and necropsied at 14 dpi. Broncho-alveolar fluid (BALF) and serum samples were collected at each of the necropsy timepoints described above. To assess virus replication in contact ferrets, nasal wash samples were collected at 0, 1, 3, 5, 7, 9, 11, and 12 days post-contact (dpc), following methods previously described [25]. BALF and plasma samples from ferrets were collected at necropsy on 12 dpc [25]. To confirm the pig-to-ferret transmission by the seroconversion of contact ferrets, the sera from contact ferrets collected at 12 dpc were analyzed by HI assay and a commercial blocking enzyme-linked immunosorbent assay (ELISA) against influenza A nucleoprotein (NP) (MultiS ELISA; Idexx, Westbrook, ME, USA) with a positive optical density (O.D.) cut-off of <0.6. 

### 2.5. Virus Replication and Shedding

Nasal swab, nasal wash, and BALF samples were titrated on MDCK cells to evaluate virus replication in the nose and lungs, as previously described for pigs [28] and ferrets [25]. MDCK-inoculated monolayers were evaluated for cytopathic effect (CPE) between 48 and 72 h post-infection, fixed with 4% phosphate-buffered formalin, and stained using immunocytochemistry (ICC) with an anti-influenza A NP monoclonal antibody [29]. A TCID_50_ titer per mL was calculated for each positive sample. Nasal wash samples that were positive in the virus isolation but negative in the virus titration were confirmed by qPCR VetMAX™-Gold SIV Detection Kit (ThermoFisher Scientific, MA, USA) and run with a qPCR standard curve included with the kit, ranging from 10–1,000,000 copies per µL.

### 2.6. Pathologic Examination

At 5 dpi, the percentage of the lung affected by purple-red consolidation typical of influenza virus in swine was visually estimated [28]. The percentage of the lung affected with purple-red consolidation typical of influenza virus in ferrets was visually estimated at 12 dpc to assess disease resolution if infected. Tissue samples from the trachea and right middle or affected lung lobe were fixed in 10% buffered formalin for histopathologic examination. Tissues were processed by routine histopathologic procedures, and slides were stained with hematoxylin and eosin (H&E) or immunohistochemistry [30].

### 2.7. Microbiological Assays

BALF samples were cultured for aerobic bacteria on blood agar and Casmin (NAD-enriched) plates to indicate the presence of concurrent bacterial pneumonia. PCR assays for porcine circovirus 2 (PCV2) were conducted for swine BALF samples [31]. To exclude other causes of pneumonia in pigs, commercial assays for *Mycoplasma hyopneumoniae* and PRRSV were conducted according to the manufacturer’s recommendations (VetMax, Life Technologies, Carlsbad, CA, USA).

### 2.8. Data Analysis

Results were analyzed with Prism 8 (GraphPad, San Diego, CA, USA) with analysis of variance (ANOVA), with *p* < 0.05 considered significant. Variables with significant effects by treatment group were subjected to pairwise mean comparisons using the Tukey-Kramer test. Clinical data associated with this study are available for download from the USDA Ag Data Commons at https://doi.org/10.15482/USDA.ADC/1528327, accessed on 5 December 2022 and the phylogenetic analyses are available from https://github.com/flu-crew/datasets, accessed on 5 December 2022.

## 3. Results

### 3.1. Genetic Characterization of Dominant U.S. Swine H3N2 Strains

**Figure 1:**
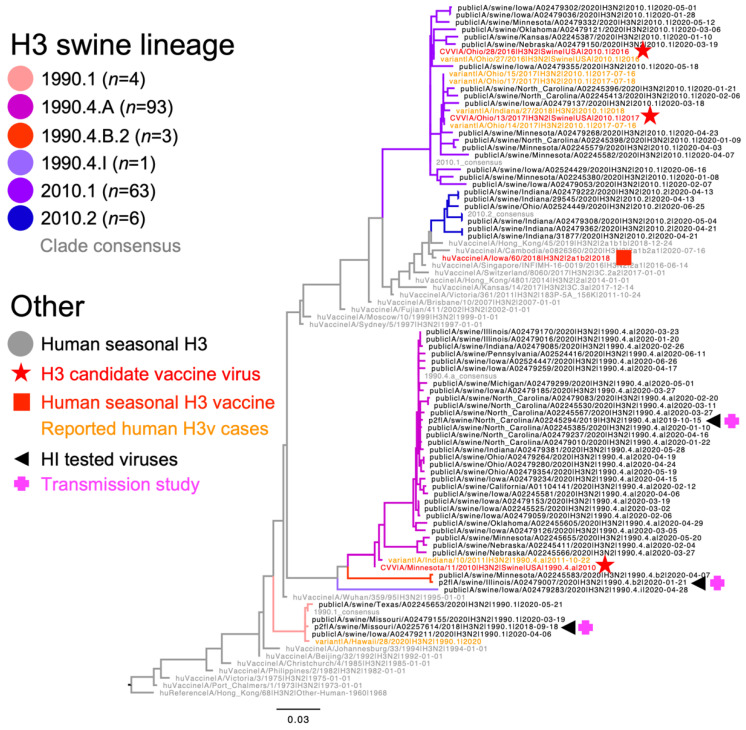
Evolutionary relationships of contemporary H3 swine influenza A viruses. A representative random sample of H3 swine hemagglutinin (HA) genes collected between January 2020 and June 2020. Reference human HA genes, candidate vaccine viruses (CVV), and variant cases are indicated by branch color or shape. Swine IAV strains tested in hemagglutination inhibition assays are marked by a black triangle, and those used in transmission studies by a pink plus sign (+). The numbers in parentheses indicate the number of each genetic clade detected during the sampling period.

There were 180 H3 HA genes collected in swine between 1 January 2020, and 30 June 2020. These HA genes represented six evolutionary lineages, eight genetic clades, and a single human-to-swine spillover: 1970.1 (*n* = 6, 3.3%), 1990.1 (*n* = 4, 2.2%), 1990.4.A (*n* = 93, 51.7.%), 1990.4.B.2 (*n* = 3, 1.7%), 1990.4.I (*n* = 1, 0.6%), 2000.3 (*n* = 3, 1.7%), 2010.1 (*n* = 63, 35%), 2010.2 (*n* = 6, 3.3%), and Other-Human-1990 (*n* = 1, 0.6%). Figure 1 shows the evolutionary relationships of the selected contemporary representative H3N2 and the most similar CVV or a human seasonal H3N2 vaccine and if a variant case occurred. We excluded the globally detected 1970.1, 2000.3, and Other-Human-1990 lineages from further analyses as they were not detected in U.S. swine. The genome constellations of internal genes have different lineage designations, “T” for the TRIG lineage, “P” for the H1N1pdm09 lineage, and “V” for the lineage derived from a swine live attenuated influenza virus vaccine (LAIV). The selected H3N2 viruses have different genome constellations: The A/swine/Missouri/A02257614/2018 strain has the HA gene from the 1990.1 swine LAIV origin lineage, the NA gene from the 1998-N2 swine LAIV origin, and the internal gene constellation is TVVVPT in the order of PB2, PB1, PA, NP, M, and NS gene segments. The HA segment of this virus has 52 amino acid residues different from HuVac A/Iowa/60/2018. The A/swine/North Carolina/A02245294/2019 strain has the HA from 1990.4.A lineage, NA from the 2002-N2 lineage, and an internal gene constellation of TTTPPT. The HA segment of this virus has nine residues different from the within-clade CVV A/Minnesota/11/2010. The A/swine/Illinois/A02479007/2020 strain has the HA from the 1990.4.B.2 lineage, NA from the 2002-N2 lineage, and internal genes of TTPTPT. The HA segment of this virus has 27 residues different from the CVV A/Minnesota/11/2010. The amino acid differences between the representative HA gene and the within-clade CVV, or human seasonal vaccine, are displayed in Tables S1–S4.

### 3.2. Loss in Cross-Reactivity between Dominant Swine H3N2 Strains and CVV or HuVac

The HA1 domain of selected representative swine H3N2 strains was compared to the HuVac and CVV vaccine strains. A range in percentages of amino acid identity was observed (Table 1). To determine if CVV or HuVac antisera had cross-reactivity against contemporary swine H3N2 strains, we tested the representative U.S. dominant swine H3N2 strains against reference ferret antisera generated against CVVs from the same genetic clade, or the most genetically similar CVV if there was no within-clade CVV. Ferret antiserum tested with the clade 1990.1 representative strain, A/swine/Missouri/A02257614/2018, demonstrated a 32-fold decrease in HI titer from the most similar clade 1990.4.A CVV, A/Minnesota/11/2010, and a >32-fold decrease from the HuVac A/Iowa/60/2018. Ferret antiserum tested with this strain had a 32-fold decrease from H3N2v A/Indiana/27/2018, a representative of contemporary swine strains. Antiserum was tested with the representative strain of clade 1990.4.A A/swine/North Carolina/A02245294/2019 demonstrated a 16-fold-decrease in HI titer compared to the within-clade CVV A/Minnesota/11/2010 and a greater than 32-fold decrease from the HuVac A/Iowa/60/2018. Antiserum tested with this strain also had a 32-fold decrease from the H3N2v virus, A/Indiana/27/2018. The 1990.4.B.2 clade represented by A/swine/Illinois/A02479007/2020 lacks a CVV, and ferret antiserum from the most similar CVV, A/Minnesota/11/2010, demonstrated a 32-fold-decrease and 16-fold decrease with the HuVac A/Iowa/60/2018 antiserum, respectively. The H3N2v A/Indiana/27/2018 antiserum had a 64-fold decrease with this strain. None of the selected representative swine strains cross-reacted with the IDCDC-RG55C A/Ohio/28/2016-like CVV. These data demonstrated that contemporary representative H3N2 viruses from the 1990.1, 1990.4.A, and 1990.4.B.2 clades frequently detected in U.S. pig populations had limited cross-reactivity against HuVac and CVV sera (Table 2).

### 3.3. Swine-to-Ferret Transmission of Antigenically Drifted Swine H3N2 Lineages

**Figure 2:**
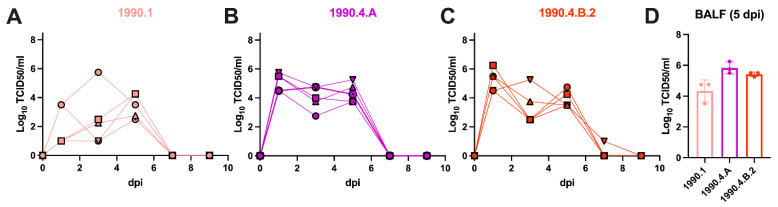
Infected pig nasal virus titers and BALF titers. Nasal shedding of H3N2 clades: 1990.1 (**A**), 1990.4.A (**B**), 1990.4.B.2 (**C**) was measured on 1, 3, 5, 7 and 9 dpi, and BALF (**D**) were collected at 5 dpi. Nasal swab samples and BALF were measured by TCID_50_ in MDCK cells and recorded as log_10_ TCID_50_/mL.

Pigs infected with the 1990.1 clade virus demonstrated a profile of modest shedding with group mean titers of 1.78 × 10^2^ TCID_50_/mL at 1 dpi and reached a peak group mean titer of 3.16 × 10^3^ TCID_50_/mL at 5 dpi (Figure 2A). In contrast, pigs infected with the 1990.4.A clade virus demonstrated high virus shedding with group mean titers of 1.78 × 10^5^ TCID_50_/mL at 1 dpi and 2.23 × 10^4^ TCID_50_/mL at 5 dpi (Figure 2B). Similarly, pigs infected with the 1990.4.B.2 clade virus shed high virus titers at 1 dpi with group mean titers of 1.78 × 10^5^ TCID_50_/mL, 1.78 × 10^3^ TCID_50_/mL at 3 dpi, and shed virus until 5 dpi with a group mean titer of 1.78 × 10^4^ TCID_50_/mL. Only one of the remaining pigs was infected with the 1990.4.B.2 virus was positive in nasal swabs at 7 dpi (Figure 2C). The other remaining pigs from all groups had no detectable virus in nasal swabs after 5 dpi. Pigs in all groups had virus detected in the lungs at 5 dpi, with group mean titers of 1.78 × 10^4^ TCID_50_/mL for the clade 1990.1 virus, 5.62 × 10^5^ TCID_50_/mL for the clade 1990.4.A virus and 3.16 × 10^5^ TCID_50_/mL for the clade 1990.4.B.2 virus (Figure 2D), but macroscopic and microscopic lesions in the lungs were minimal in all groups (Table S5). The two remaining pigs from each group seroconverted at 14 dpi with HI titers ranging from 160–320, confirming infection (Table S6).

**Figure 3:**
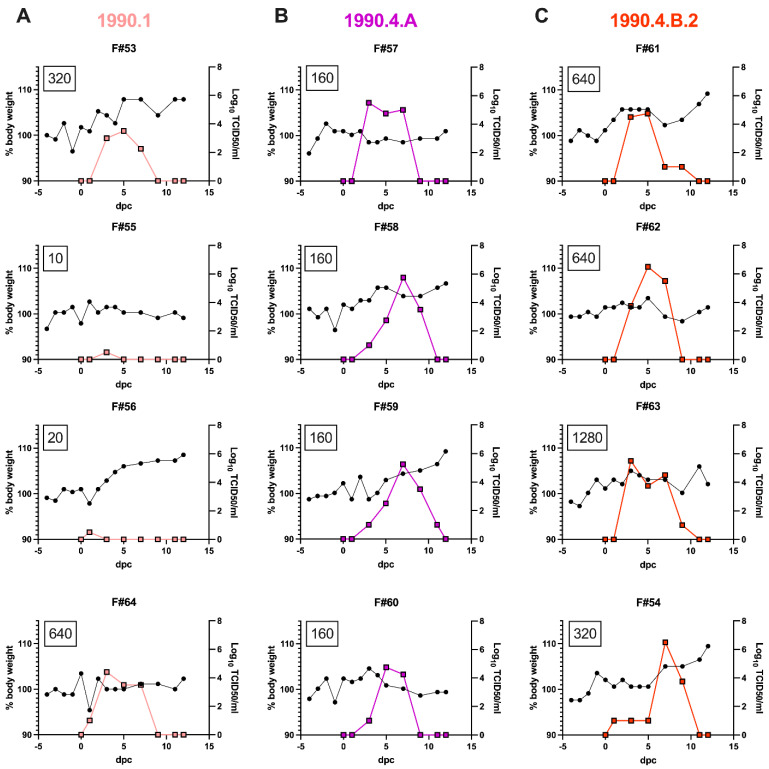
Swine H3N2 transmitted to ferrets. Percent body weight change and nasal wash virus titers were recorded for individual ferrets exposed to 1990.1 (**A**), 1990.4.A (**B**), and 1990.4.B.2 (**C**) swine H3N2 strains. Ferret body weight was taken daily and recorded as a percentage of the −3 day average body weight prior to exposure (left axis, black circles). Nasal washes were measured for viral shedding by TCID_50_ in MDCK cells and recorded as log_10_ TCID_50_/mL (right axis, color square). The black boxed numbers indicate the HI titers of sera collected at 12 days post contact (dpc).

Two out of four contact ferrets from the 1990.1 group started shedding virus at low levels, with an average of 10^1^ TCID_50_/mL in nasal washes at 1 dpc and at 3 dpc and an average titer of 5.62 × 10^2^ TCID_50_/mL (Table 3, Figure 3). Two out of four contact ferrets had peak virus shedding at 5 dpc with an average titer of 3.16 × 10^3^ TCID_50_/mL and continued to shed virus until 7 dpc with 5.62 × 10^2^ TCID_50_/mL. Detection of virus by cell culture in the nasal washes of F#55 at 3 dpc and F#56 at 1 dpc was confirmed by qPCR, with C_t_ values of 36, approximately 100 copies/μL. This may represent surface contamination of their noses rather than a productive infection since the virus was not detected on other days and these two ferrets remained seronegative. Only two out of four contact ferrets of the 1990.1 clade seroconverted by 1 dpc. F#55 and F#56 had HI titers below 40 and were also negative by NP-ELISA (Table 3, Figure 3A).

All four contact ferrets of the 1990.4.A group started shedding virus at 3 dpc with an average titer of 1.78 × 10^2^ TCID_50_/mL. Two out of four ferrets peaked at 7 dpc (F#58 and F#59), one peaked at 3 dpc (F#57), and another peaked at 5 dpc (F#60). The virus titer group average was 5.62 × 10^3^ TCID_50_/mL at 5 dpc and 1.78 × 10^5^ TCID_50_/mL at 7 dpc. Two out of four contact ferrets continued shedding virus with an average titer of 3.16 × 10^3^ TCID_50_/mL at 9 dpc, and only one ferret shed virus until 11 dpc at 10^1^ TCID_50_/mL. All four contact ferrets from the 1990.4.A group seroconverted by 12 dpc (Table 3, Figure 3B).

One out of four contact ferrets of 1990.4.B.2 group started shedding virus at 1 dpc with a titer of 10^1^ TCID_50_/mL. All four contact ferrets shed virus at 3 dpc with an average titer of 5.62 × 10^3^ TCID_50_/mL, increased virus titers at 5 dpc with an average titer of 1.78 × 10^4^ TCID_50_/mL and maintained virus shedding at 7 dpc with an average titer of 3.16 × 10^4^ TCID_50_/mL. Then, after the virus peak, three out of four contact ferrets had lower virus shedding, with an average titer of 1.78 × 10^2^ TCID_50_/mL until 9 dpc. At 12 dpc, no virus was detected in nasal washes or BALF samples from ferrets. All four contact ferrets from the 1990.4.B.2 group seroconverted by 12 dpc (Table 3, Figure 3C).

There were minimal clinical signs in ferrets, with the exception of one ferret (F#54) from the 1990.4.B.2 group, in which coughing was observed and it had visible lung lesions at 12 dpc. None of the contact ferrets from the 1990.1 group demonstrated a febrile response at any time. In the 1990.4.A group, only F#59 displayed a febrile response of 39.6 °C at 9 and 12 dpc. In the 1990.4.B.2 clade group, F#54 displayed a febrile response at 4 dpc of 39.7 °C. Body weight change was not significantly different between groups; however, ferrets positive for virus in nasal washes showed a trend toward static weight during the virus shedding period (Figure 3).

## 4. Discussion

Swine IAVs are considered a threat to public health due to swine populations worldwide harboring a vast array of antigenically diverse IAVs that occasionally spill over into humans. Consequently, swine-origin variant virus cases in humans have raised public health concerns, and there is a critical need to assess the zoonotic potential of these viruses before swine-to-human epidemics or pandemics occur [32]. Zoonotic interspecies IAV transmission requires important factors, such as virus adaptation, exposure of a susceptible human, virus load, and close contact with infected swine that increases the risk of infection [33,34,35,36]. The US CDC have reported variant cases since 2005, with most cases identified in individuals (mainly children) at swine exhibitions who had close contact with swine at agricultural fairs. Within this period, H3N2v virus infections have been the most frequently detected in the U.S. [32]. Although person-to-person transmission of variant viruses is rare, variants have the potential to evolve and may acquire the ability to transmit from human-to-human, as occurred with the H1N1pdm09 [37]. In response to the recognition of the public health risk of swine IAV, human pandemic preparedness efforts have expanded to include the characterization of swine-origin variant strains and, when appropriate, the development of CVV. However, the genetic and antigenic diversity of swine H3 clades [18,38] requires regular characterization to identify swine IAV that represent a pandemic threat. Here, we identified U.S. swine H3N2 lineages with zoonotic potential based on the loss of cross-reactivity of post-infection ferret antisera raised to relevant CVV or human seasonal vaccines with an integrated assessment using an interspecies swine-to-ferret transmission model.

The 1990.1, 1990.4.A, and 1990.4.B.2 clades characterized in this study currently circulate in U.S. pig populations. The 1990.4.A clade had a significant increase in detection frequency from 7% in 2017 to 32% in 2019 [38]. Since 2019, the 1990.4.A clade has been one of the most frequent IAV lineages detected in the U.S. swine population, comprising ~52% of detections between 2020 and 2022 [39]. Although there is a within-clade CVV (A/Minnesota/11/2010 × 203), a contemporary representative strain of the 1990.4.A clade displayed a 16-fold reduction in HI cross-reactivity, suggesting a loss in protection and potential zoonotic risk. The other two swine H3N2 strains (1990.1 and 1990.4.B.2) represented 2.9% and 3% of detections in the U.S. in 2020, respectively. Although these two clades were infrequently detected, there have been sustained detections over the past decade, and there are no available CVVs within these clades. Additionally, our serologic assessment indicated very limited cross-reactivity to an available CVV and/or human seasonal vaccines. Given limited cross-reactivity observed in our study, the 1990.1 and 1990.4.B.2 are not covered by existing pandemic preparedness CVVs or human seasonal vaccines. Additionally, in a previous study, 1990.1, 1990.4.A, and 1990.4.B strains demonstrated limited cross-reactivity to post-infection and post-vaccination adult human cohort sera, with results suggesting that older subjects have reduced immunity to these swine IAV clades [18]. Consequently, we selected contemporary strains from these clades to assess zoonotic potential in an interspecies transmission model.

To determine the potential for interspecies transmission of the selected H3N2 swine viruses, we used a swine-to-ferret transmission model [25,40]. Ferrets are widely used as an animal model for pathogenesis and transmission studies of influenza viruses and vaccine efficacy studies and are a useful animal model for humans because of their anatomic and physiological similarities [41]. Both 1990.4.A and 1990.4.B.2 clades replicated efficiently in the respiratory tract of pigs and transmitted high virus titers in nasal washes to contact ferrets, as confirmed by seroconversion of contact ferrets at 12 dpc. A previous ferret-to-ferret transmission study with an H3N2v virus demonstrated that high virus shedding titers in ferret nasal washes correlated with high growth in human airway epithelial cells, suggesting that these viruses can infect cells of the human airway [42]. Although the body weight and temperature were not significantly different in ferrets infected with all the viruses we tested, the 1990.4.A and 1990.4.B.2 contact groups displayed a trend of weight loss or no weight gain when virus shedding was detected. In contrast, the 1990.1 strain demonstrated a lower profile of virus shedding and transmission, as demonstrated by the absence of seroconversion in two of the four contact ferrets and low virus titers in nasal shedding. Although two ferrets did not seroconvert by 12 dpc, transient virus in their nasal cavities was detected at 1 dpc and 3 dpc, confirmed by qPCR. Because this study was focused on transmission, we did not characterize lung lesions or viruses in the lungs in ferrets until necropsy by 12 dpc, reflecting illness recovery by that timepoint. Only one of the ferrets infected with 1990.4.B.2 presented clinical respiratory signs, such as coughing and dyspnea, and displayed lung lesions at the necropsy on 12 dpc. A previous swine-to-ferret study of a 2010.1 lineage of H3N2 demonstrated efficient interspecies transmission [25]. Viruses from this 2010.1 swine lineage have resulted in 73 variant cases with genetic sequence data available between 2010 and 2022 [25,32]. Our data suggest that H3 viruses introduced to US swine in the 1990s (1990.4.A and 1990.4.B.2) and those introduced in the 2010s (2010.1) have retained the ability to transmit and replicate in humans and have zoonotic potential.

Although HA plays a key role in the restriction of interspecies transmission, efficient virus infection and transmission require balanced actions of HA receptor-binding and NA sialidase activity [43]. The HA protein of IAV is the primary target of protective immune responses and is a major component of vaccines. Thus, HA cross-neutralizing antibodies are important for a protective immune response. Substitutions in seven amino acid positions near the receptor binding site of the HA (145, 155, 156, 158, 159, and 189; H3 numbering) are key for antigenic drift in human [44,45] and swine IAV [12], and determine the antigenic phenotype of the virus. Our data support the proposition that genetic changes (Table 1 and Tables S1–S4) in the HA1 for the 1990.1, 1990.4.A, and 1990.4.B.2 swine clades resulted in significant antigenic drift from the human-seasonal vaccines and the tested CVVs.

Reassortant swine IAV with different gene constellations have demonstrated different profiles of transmission efficiency in pigs [46,47]. The 1990.1 isolate selected for this study contained PB1, PA, and NP genes derived from a commercial live attenuated influenza vaccine (LAIV) for swine (Boehringer Ingelheim, St. Joseph, MO, USA) [48,49]. Reassortment between the LAIV vaccine and endemic swine IAV field strains circulating in the U.S. was detected in 2018 [50] and vaccine use was discontinued. The 1990.1 virus displayed a lower virus shedding profile and a lower number of contact ferret seroconversions by 12 dpc compared to the 1990.4.A and 1990.4.B.2 groups. Despite differences in genome constellation, all three clades from the 1990s (1990.1, 1990.4.A, and 1990.4.B.2) contained the M segment from the H1N1pdm09 lineage, which has previously been associated with high transmission efficiency in a guinea pig model [51]. Notably, variant 1990.4.A H3N2 viruses detected in 2011–2012 contained the M from the pandemic and the rest of the internal genes from the TRIG lineage [52]. PB2 and PA segments are important components of the viral polymerase complex and essential for viral replication. The viruses from genetic clades represented in this study, 1990.1, 1990.4.A, and 1990.4.B.2, contained residues encoded in the PB2 gene previously described to enhance replication in humans: 271A, 590S, 591R, and 661A, as well as the residue 669V encoded in the PA gene [53,54,55,56]. These genetic markers support the proposition that these swine viruses represent an increased zoonotic risk.

Taken together, our study indicated that the swine H3 1990.1, 1990.4.A, and 1990.4.B.2 clades are a zoonotic risk as they displayed reduced cross-reactivity with ferret antisera raised to human seasonal vaccines and/or CVVs and transmitted from pig-to-ferrets. Our findings suggest that existing CVVs should be updated to reflect contemporary swine IAV diversity; specifically, we suggest that the 1990.4.A clade that is widespread in U.S. swine populations and that has previously caused a significant number of H3 variant human cases requires revision. In addition, our study suggests that minor swine IAV clades that are regularly detected in US swine populations have zoonotic potential and should be considered in risk assessments of swine H3N2 IAV for pandemic preparedness strategies.

## Figures and Tables

**Table 1:** Pairwise amino acid sequence similarity of the HA1 domain from swine H3 clade consensus sequences from the U.S. to candidate vaccine viruses or human seasonal vaccine viruses and clade representative viruses used in this study. Within-clade comparisons are highlighted in grey.

	1990.1 Consensus	A/swine/Missouri/A02257614/2018	1990.4.A Consensus	A/Minnesota/11/2010 CVV	A/swine/North Carolina/A02245294/2019	1990.4.B.2. Consensus	A/swine/Illinois/A02479007/2020	2010.1 Consensus	A/Ohio/28/2016	A/Indiana/27/2018 *	A/Iowa/60/2018 HuVac
1990.1 consensus		99.70	85.71	87.23	85.71	87.23	86.02	84.19	84.19	83.89	83.89
A/swine/Missouri/A02257614/2018	99.70		86.02	87.54	86.02	87.54	86.32	84.19	84.19	83.89	84.19
1990.4.A consensus	85.71	86.02		97.26	100	90.88	89.67	83.59	82.98	82.37	81.46
A/Minnesota/11/2010 CVV	87.23	87.54	97.26		97.26	93.01	91.79	83.89	83.89	82.98	83.28
A/swine/North Carolina/A02245294/2019	85.71	86.02	100	97.26		90.88	89.67	83.59	82.98	82.37	81.46
1990.4.B.2 consensus	87.23	87.54	90.88	93.01	90.88		95.14	84.5	85.11	82.98	83.89
A/swine/Illinois/A02479007/2020	86.02	86.32	89.67	91.79	89.67	95.14		82.98	83.28	82.37	82.67
2010.1 consensus	84.19	84.19	83.59	83.89	83.59	84.5	82.98		99.39	98.18	89.36
A/Ohio/28/2016	84.19	84.19	82.98	83.89	82.98	85.11	83.28	99.39		97.57	89.67
A/Indiana/27/2018 *	83.89	83.89	82.37	82.98	82.37	82.98	82.37	98.18	97.57		88.15
A/Iowa/60/2018 HuVac	83.89	84.19	81.46	83.28	81.46	83.89	82.67	89.36	89.67	88.15	

* A/Indiana/27/2018 H3N2v is a variant strain representing contemporary 2010.1 swine strains.

**Table 2:** Hemagglutination inhibition assays with ferret antisera raised against CVV and vaccine viruses tested for the ability to inhibit hemagglutination of contemporary swine viruses.

Strain	Lineage	Antigenic Motif	A/Minnesota/11/2010 × 203	IDCDC-RG55C A/Ohio/28/2016-like	A/Indiana/27/2018 *	A/Iowa/60/ 2018
A/swine/Missouri/A02257614/2018	1990.1	KHKEYS	40	<10	20	<10
**A/Minnesota/11/2010 × 203**	1990.4.A	NYNNYK	**1280**	<10	10	<10
A/swine/North Carolina/A02245294/2019	1990.4.A	NYHNYK	80	<10	20	<10
A/swine/Illinois/A02479007/2020	1990.4.B.2	SYHNYK	40	<10	10	20
**IDCDC-RG55C A/Ohio/28/2016-like**	2010.1	KTHNFK	<10	**1280**	80	20
A/Indiana/27/2018 *	2010.1	NTRDFT	<10	10	**640**	10
**A/Iowa/60/2018**	HuVac	STHNYK	<10	<10	10	**320**

* A/Indiana/27/2018 H3N2v is representative of contemporary 2010.1 swine strains. Vaccine strains and homologous titers are bolded; gray highlighted cells indicate the within-clade titer of a contemporary swine strain.

**Table 3:** Cumulative clinical, viral, and serological measures of ferrets exposed to H3N2-infected pigs.

Viral Clade	Ferret #	Change in Bodyweight (%) from 0–12 dpc	dpc with Nasal Detection	Peak Titer log_10_ TCID_50_/mL	12 dpc HI Titer	NP-ELISA S/N
1990.1	53	7.9	3,5,7	3.5	320	0.171
	55	12.1	3	0.5	10	1.003
	56	9.4	1	0.5	20	1.116
	64	3.5	1,3,5,7	4.4	640	0.188
1990.4.A	57	4.9	3,5,7	5.5	160	0.335
	58	5.5	3,5,7,9	5.8	160	0.314
	59	10.5	3,5,7,9,11	5.2	160	0.410
	60	1.5	3,5,7	4.8	160	0.272
1990.4.B.2	61	10.3	3,5,7,9	4.8	640	0.213
	62	2.0	3,5,7	6.5	640	0.265
	63	3.9	3,5,7,9	5.5	1280	0.511
	54	11.8	1,3,5,7,9	6.5	320	0.555
No virus	13	5.2	none	0	<10	1.082
	14	3.5	none	0	<10	0.829

## Data Availability

**The** clinical data associated with this study are available for download from the USDA Ag Data Commons at https://doi.org/10.15482/USDA.ADC/1528327 (Accessed on 7 December 2022) and the phylogenetic analyses are available at https://github.com/flu-crew/datasets (Accessed on 7 December 2022).

## Supplementary Materials

The following supporting information can be downloaded at: https://www.mdpi.com/article/10.3390/v15020331/s1, Table S1: Amino acid differences between human seasonal vaccine A/Iowa/60/2018 and the 1990.1 clade represented by A/swine/Missouri/A02257614/2018 strain. Annotations include mutations within putative epitopes and receptor binding sites; Table S2: Amino acid differences between A/Minnesota/11/2010 CVV and the 1990.4.A is the clade represented by A/swine/North Carolina/A02245294/2019 and the 1990.4.B.2 is the clade represented by A/swine/Illinois/A02479007/2020. Annotations include mutations within putative epitopes and receptor binding sites; Table S3: Amino acid differences between A/Ohio/28/2016 CVV and the 2010.1 clade represented by the A/Indiana/27/2018 variant strain. Annotations include mutations within putative epitopes and receptor binding sites; Table S4: Amino acid differences between A/Ohio/28/2016 CVV and A/Iowa/60/2018 HuVac. Annotations include mutations within the putative epitope and receptor binding site; Table S5: Percentage of macroscopic lung lesions and microscopic lung and trachea scores in pigs; Table S6: Pig HI data at 0, 5, and 14 dpi; Figure S1: Detection proportions of swine H3N2 collected in 2020 in the USDA influenza A virus in swine surveillance system.

## References

[1] Jester B.J., Uyeki T.M., Jernigan D.B. (2020). Fifty Years of Influenza A(H3N2) Following the Pandemic of 1968. Am. J. Public Health.

[2] Long J.S., Mistry B., Haslam S.M., Barclay W.S. (2019). Publisher Correction: Host and viral determinants of influenza A virus species specificity. Nat. Rev. Microbiol..

[3] Garten R.J., Davis C.T., Russell C.A., Shu B., Lindstrom S., Balish A., Sessions W.M., Xu X., Skepner E., Deyde V. (2009). Antigenic and Genetic Characteristics of Swine-Origin 2009 A(H1N1) Influenza Viruses Circulating in Humans. Science.

[4] Olsen C.W., Karasin A.I., Carman S., Li Y., Bastien N., Ojkic D., Alves D., Charbonneau G., Henning B.M., Low D.E. (2006). Triple Reassortant H3N2 Influenza A Viruses, Canada, 2005. Emerg. Infect. Dis..

[5] Rajao D.S., Vincent A.L., Perez D. (2018). Adaptation of Human Influenza Viruses to Swine. Front. Veter- Sci..

[6] Zhou N.N., Senne D.A., Landgraf J.S., Swenson S.L., Erickson G., Rossow K., Liu L., Yoon K., Krauss S., Webster R.G. (1999). Genetic reassortment of avian, swine, and human influenza A viruses in American pigs. J. Virol..

[7] Webby R.J., Swenson S.L., Krauss S.L., Gerrish P.J., Goyal S.M., Webster R.G. (2000). Evolution of Swine H3N2 Influenza Viruses in the United States. J. Virol..

[8] Nelson M.I., Vincent A.L., Kitikoon P., Holmes E.C., Gramer M.R. (2012). Evolution of Novel Reassortant A/H3N2 Influenza Viruses in North American Swine and Humans, 2009–2011. J. Virol..

[9] Vincent A.L., Ma W., Lager K.M., Janke B.H., Richt J.A. (2008). Swine influenza viruses a North American perspective. Adv. Virus Res..

[10] Anderson T.K., Chang J., Arendsee Z.W., Venkatesh D., Souza C.K., Kimble J.B., Lewis N.S., Davis C.T., Vincent A.L. (2020). Swine Influenza A Viruses and the Tangled Relationship with Humans. Cold Spring Harb. Perspect. Med..

[11] Bolton M.J., Abente E.J., Venkatesh D., Stratton J.A., Zeller M., Anderson T.K., Lewis N.S., Vincent A.L. (2018). Antigenic evolution of H3N2 influenza A viruses in swine in the United States from 2012 to 2016. Influ. Other Respir. Viruses.

[12] Lewis N.S., Anderson T.K., Kitikoon P., Skepner E., Burke D.F., Vincent A.L. (2014). Substitutions near the Hemagglutinin Receptor-Binding Site Determine the Antigenic Evolution of Influenza A H3N2 Viruses in U.S. Swine. J. Virol..

[13] Rajão D.S., Gauger P.C., Anderson T.K., Lewis N.S., Abente E.J., Killian M.L., Perez D.R., Sutton T.C., Zhang J., Vincent A.L. (2015). Novel Reassortant Human-Like H3N2 and H3N1 Influenza A Viruses Detected in Pigs Are Virulent and Antigenically Distinct from Swine Viruses Endemic to the United States. J. Virol..

[14] Sharma A., Zeller M.A., Souza C.K., Anderson T.K., Vincent A.L., Harmon K., Li G., Zhang J., Gauger P.C. (2022). Characterization of a 2016–2017 Human Seasonal H3 Influenza A Virus Spillover Now Endemic to U.S. Swine. Msphere.

[15] Zeller M., Li G., Harmon K.M., Zhang J., Vincent A.L., Anderson T.K., Gauger P.C. (2018). Complete Genome Sequences of Two Novel Human-Like H3N2 Influenza A Viruses, A/swine/Oklahoma/65980/2017 (H3N2) and A/Swine/Oklahoma/65260/2017 (H3N2), Detected in Swine in the United States. Genome Announc..

[16] Robertson J.S., Nicolson C., Harvey R., Johnson R., Major D., Guilfoyle K., Roseby S., Newman R., Collin R., Wallis C. (2011). The development of vaccine viruses against pandemic A(H1N1) influenza. Vaccine.

[17] Burke S.A., Trock S.C. (2018). Use of Influenza Risk Assessment Tool for Prepandemic Preparedness. Emerg. Infect. Dis..

[18] Souza C.K., Anderson T.K., Chang J., Venkatesh D., Lewis N.S., Pekosz A., Shaw-Saliba K., Rothman R.E., Chen K.-F., Vincent A.L. (2022). Antigenic Distance between North American Swine and Human Seasonal H3N2 Influenza A Viruses as an Indication of Zoonotic Risk to Humans. J. Virol..

[19] Shu Y., McCauley J. (2017). GISAID: Global initiative on sharing all influenza data—From vision to reality. Euro Surveill.

[20] Katoh K., Standley D.M. (2013). MAFFT Multiple Sequence Alignment Software Version 7: Improvements in Performance and Usability. Mol. Biol. Evol..

[21] Hoang D.T., Chernomor O., Von Haeseler A., Minh B.Q., Vinh L.S. (2018). UFBoot2: Improving the Ultrafast Bootstrap Approximation. Mol. Biol. Evol..

[22] Minh B.Q., Schmidt H.A., Chernomor O., Schrempf D., Woodhams M.D., von Haeseler A., Lanfear R. (2020). IQ-TREE 2: New Models and Efficient Methods for Phylogenetic Inference in the Genomic Era. Mol. Biol. Evol..

[23] Arendsee Z.W., Baker A.L.V., Anderson T.K. (2022). smot: A python package and CLI tool for contextual phylogenetic subsampling. J. Open Source Softw..

[24] Chang J., Anderson T.K., Zeller M., Gauger P.C., Vincent A.L. (2019). octoFLU: Automated Classification for the Evolutionary Origin of Influenza A Virus Gene Sequences Detected in U.S. Swine. Microbiol. Resour. Announc..

[25] Kaplan B.S., Kimble J.B., Chang J., Anderson T.K., Gauger P.C., Janas-Martindale A., Killian M.L., Bowman A.S., Vincent A.L. (2020). Aerosol Transmission from Infected Swine to Ferrets of an H3N2 Virus Collected from an Agricultural Fair and Associated with Human Variant Infections. J. Virol..

[26] Kitikoon P., Gauger P.C., Vincent A.L. (2014). Hemagglutinin Inhibition Assay with Swine Sera. Methods Mol. Biol..

[27] Reed L.J., Muench H. (1938). A simple method of estimating fifty per cent endpoints. Am. J. Epidemiol..

[28] Gauger P.C., Vincent A.L., Loving C.L., Henningson J.N., Lager K.M., Janke B.H., Kehrli M.E., Roth J.A. (2012). Kinetics of Lung Lesion Development and Pro-Inflammatory Cytokine Response in Pigs with Vaccine-Associated Enhanced Respiratory Disease Induced by Challenge with Pandemic (2009) A/H1N1 Influenza Virus. Vet. Pathol..

[29] Kitikoon P., Nilubol D., Erickson B.J., Janke B.H., Hoover T.C., Sornsen S.A., Thacker E.L. (2006). The immune response and maternal antibody interference to a heterologous H1N1 swine influenza virus infection following vaccination. Vet. Immunol. Immunopathol..

[30] Vincent L.L., Janke B.H., Paul P.S., Halbur P.G. (1997). A Monoclonal-Antibody-Based Immunohistochemical Method for the Detection of Swine Influenza Virus in Formalin-Fixed, Paraffin-Embedded Tissues. J. Vet. Diagn. Investig..

[31] Opriessnig T., Yu S., Gallup J.M., Evans R.B., Fenaux M., Pallares F., Thacker E.L., Brockus C.W., Ackermann M.R., Thomas P. (2003). Effect of Vaccination with Selective Bacterins on Conventional Pigs Infected with Type 2 Porcine Circovirus. Vet. Pathol..

[32] Centers for Disease Control and Prevention (CDC) Reported Infections with Variant Influenza Viruses in the United States. https://www.cdc.gov/flu/swineflu/variant-cases-us.htm.

[33] Henritzi D., Petric P.P., Lewis N.S., Graaf A., Pessia A., Starick E., Breithaupt A., Strebelow G., Luttermann C., Parker L.M.K. (2020). Surveillance of European Domestic Pig Populations Identifies an Emerging Reservoir of Potentially Zoonotic Swine Influenza A Viruses. Cell Host Microbe.

[34] Kessler S., Harder T.C., SchwemmLe M., Ciminski K. (2021). Influenza A Viruses and Zoonotic Events-Are We Creating Our Own Reservoirs?. Viruses.

[35] Nelson M.I., Worobey M. (2018). Origins of the 1918 Pandemic: Revisiting the Swine “Mixing Vessel” Hypothesis. Am. J. Epidemiol..

[36] Short K.R., Richard M., Verhagen J.H., van Riel D., Schrauwen E.J., Brand J.M.V.D., Mänz B., Bodewes R., Herfst S. (2015). One health, multiple challenges: The inter-species transmission of influenza A virus. One Health.

[37] Novel Swine-Origin Influenza A (H1N1) Virus Investigation Team (2009). Emergence of a Novel Swine-Origin Influenza A (H1N1) Virus in Humans. N. Engl. J. Med..

[38] Neveau M.N., Zeller M.A., Kaplan B.S., Souza C.K., Gauger P.C., Vincent A.L., Anderson T.K. (2022). Genetic and Antigenic Characterization of an Expanding H3 Influenza A Virus Clade in U.S. Swine Visualized by Nextstrain. Msphere.

[39] Zeller M., Anderson T.K., Walia R.W., Vincent A.L., Gauger P.C. (2018). ISU FLUture: A veterinary diagnostic laboratory web-based platform to monitor the temporal genetic patterns of Influenza A virus in swine. BMC Bioinform..

[40] Kimble J.B., Souza C.K., Anderson T.K., Arendsee Z.W., Hufnagel D.E., Young K.M., Lewis N.S., Davis C.T., Thor S., Baker A.L.V. (2022). Interspecies Transmission from Pigs to Ferrets of Antigenically Distinct Swine H1 Influenza A Viruses with Reduced Reactivity to Candidate Vaccine Virus Antisera as Measures of Relative Zoonotic Risk. Viruses.

[41] DiPiazza A.T., Richards K.A., Liu W.-C., Albrecht R.A., Sant A.J. (2018). Analyses of Cellular Immune Responses in Ferrets following Influenza Virus Infection. Methods Mol. Biol..

[42] Pearce M.B., Jayaraman A., Pappas C., Belser J.A., Zeng H., Gustin K.M., Maines T.R., Sun X., Raman R., Cox N.J. (2012). Pathogenesis and transmission of swine origin A(H3N2)v influenza viruses in ferrets. Proc. Natl. Acad. Sci. USA.

[43] Neumann G., Kawaoka Y. (2006). Host range restriction and pathogenicity in the context of influenza pandemic. Emerg. Infect. Dis..

[44] Koel B.F., Burke D.F., Bestebroer T.M., van der Vliet S., Zondag G.C.M., Vervaet G., Skepner E., Lewis N.S., Spronken M.I.J., Russell C.A. (2013). Substitutions Near the Receptor Binding Site Determine Major Antigenic Change during Influenza Virus Evolution. Science.

[45] Burke D.F., Smith D.J. (2014). A Recommended Numbering Scheme for Influenza A HA Subtypes. PLoS ONE.

[46] Ma J., Shen H., Liu Q., Bawa B., Qi W., Duff M., Lang Y., Lee J., Yu H., Bai J. (2015). Pathogenicity and Transmissibility of Novel Reassortant H3N2 Influenza Viruses with 2009 Pandemic H1N1 Genes in Pigs. J. Virol..

[47] Rajão D.S., Walia R.R., Campbell B., Gauger P.C., Janas-Martindale A., Killian M.L., Vincent A.L. (2017). Reassortment between Swine H3N2 and 2009 Pandemic H1N1 in the United States Resulted in Influenza A Viruses with Diverse Genetic Constellations with Variable Virulence in Pigs. J. Virol..

[48] Genzow M., Goodell C., Kaiser T.J., Johnson W., Eichmeyer M. (2017). Live attenuated influenza virus vaccine reduces virus shedding of newborn piglets in the presence of maternal antibody. Influ. Other Respir. Viruses.

[49] Kaiser T.J., Smiley R.A., Fergen B., Eichmeyer M., Genzow M. (2019). Influenza A virus shedding reduction observed at 12 weeks post-vaccination when newborn pigs are administered live-attenuated influenza virus vaccine. Influ. Other Respir. Viruses.

[50] Sharma A., Zeller M., Li G., Harmon K.M., Zhang J., Hoang H., Anderson T.K., Vincent A.L., Gauger P.C. (2020). Detection of live attenuated influenza vaccine virus and evidence of reassortment in the U.S. swine population. J. Vet. Diagn. Investig..

[51] Chou Y.-Y., Albrecht R.A., Pica N., Lowen A.C., Richt J.A., García-Sastre A., Palese P., Hai R. (2011). The M Segment of the 2009 New Pandemic H1N1 Influenza Virus Is Critical for Its High Transmission Efficiency in the Guinea Pig Model. J. Virol..

[52] Epperson S., Jhung M., Richards S., Quinlisk P., Ball L., Moll M., Boulton R., Haddy L., Biggerstaff M., Brammer L. (2013). Human Infections with Influenza A(H3N2) Variant Virus in the United States, 2011–2012. Clin. Infect. Dis..

[53] Arai Y., Kawashita N., Daidoji T., Ibrahim M.S., Elgendy E., Takagi T., Takahashi K., Suzuki Y., Ikuta K., Nakaya T. (2016). Novel Polymerase Gene Mutations for Human Adaptation in Clinical Isolates of Avian H5N1 Influenza Viruses. PLoS Pathog..

[54] Hayashi T., Wills S., Bussey K.A., Takimoto T. (2015). Identification of Influenza A Virus PB2 Residues Involved in Enhanced Polymerase Activity and Virus Growth in Mammalian Cells at Low Temperatures. J. Virol..

[55] Liu Q., Qiao C., Marjuki H., Bawa B., Ma J., Guillossou S., Webby R.J., Richt J.A., Ma W. (2012). Combination of PB2 271A and SR polymorphism at positions 590/591 is critical for viral replication and virulence of swine influenza virus in cultured cells and in vivo. J. Virol..

[56] Sun X., Pulit-Penaloza J.A., Belser J.A., Pappas C., Pearce M.B., Brock N., Zeng H., Creager H.M., Zanders N., Jang Y. (2018). Pathogenesis and Transmission of Genetically Diverse Swine-Origin H3N2 Variant Influenza A Viruses from Multiple Lineages Isolated in the United States, 2011–2016. J. Virol..

