# Supplementary material for: Swine-to-Ferret Transmission of Antigenically Drifted Contemporary Swine H3N2 Influenza A Virus Is an Indicator of Zoonotic Risk to Humans
Source: Viruses. 2023 Jan 24;15(2):331. doi: 10.3390/v15020331 (PMC9962742; doi:10.3390/v15020331)
Supplement: Supplementary file 1 [file viruses-15-00331-s001.zip › viruses-2119027-supplementary.pdf]

Supplementary Materials

Table S1. Amino acid differences between human seasonal vaccine A/Iowa/60/2018 and the 1990.1 clade represented by A/swine/Missouri/A02257614/2018 strain. Annotations include mutations within putative epitope and receptor binding sites.

H3 Amino Acid Residue	A/Iowa/60/2018 HuVac	A/swine/Mis-souri/A02257614/2018	3.1990.1_consensus	Annotations
3	I	L	L	
9	S		R	
25	I	L	L	
33	R	Q	Q	
45	N	S	S	
48	I	T	T	
50	E	R	R	
57	Q	R	R	
62	G	K	K	
75	Q	H	H	
83	K	E	E	
88	V	I	I	
92	R	K	K	
106	A	S	S	
121	K	T	T	
124	S	D	D	
131	K	A	A	
133	N	D	D	Site A, RBS
135	T	G	G	RBS
137	S	Y	Y	Site A
138	A	S	S	
140	I	K	K	
144	S	V	V	Site A
145	S	K	K	Site A
155	T	H	H	Site B
156	H	K	K	
158	N	E	E	
160	T	K	K	
171	K	N	N	
172	E	D	D	
173	Q	K	K	
186	G	S	S	Site B
189	K	S	S	Site B, RBS
190	D	E	E	RBS
192	I	T	T	RBS
193	F	S	S	Site B, RBS

196	A	V	V	
198	S	A	A	
199	S	I	I	
202	I	V	V	
208	R	S	S	Site D
212	A	T	T	
222	R	W	W	RBS
223	I	V	V	RBS
225	D	G	G	RBS
227	P	S	S	
246	N	S	S	
262	S	N	N	
275	G	D	D	Site C
276	K	N	N	
278	K	Y	Y	Site C
307	R	K	K	
312	S	K	K	
Differences from consensus		**-**	**52**	
Differences from HuVac		**51**	**-**	

Table S2. Amino acid differences between A/Minnesota/11/2010 CVV and the 1990.4.A clade represented by A/swine/North Carolina/A02245294/2019 and 1990.4.B.2 clade represented by A/swine/Illinois/A02479007/2020. Annotations include mutations within putative epitope and receptor binding sites.

H3 Amino Acid Residue	A/Minnesota/11/2010 CVV	A/swine/North Carolina/A02245294/2019	A/swine/Illinois/A02479007/2020	1990.4.A_consensus	1990.4.B.2_consensus	Annotations
27	K		R			
53	N		S		S	Site C
56	H		Y			
57	Q		K		K	
83	E		K		K	
96	N	S		S		
106	A		T			
107	T		S		S	
117	N		T		T	
119	E	K		K		
121	T		N		N	
124	S	I		I		
131	A	T		T		
137	Y		I		N	Site A
138	A	S		S		
140	R		K			
142	G				E	
144	V		D		N	Site A
145	N		S		K	Site A
156	N	H	H	H	H	
159	Y				N	
163	E		A		A	
164	Q		L		L	
193	N				Y	Site B, RBS
199	S		Q		P	
203	I				T	
204	V		I		I	
207	K	R		R		Site D
216	N		D			
217	I		V		V	Site D
236	I		V			
242	I		M		M	
252	I		V			
273	H		P		P	
276	E		N		N	
278	N		S			Site C
289	P	S		S		
299	K		R		R	
323	V	I		I		
Differences from consensus		**-**	**-**	**9**	**23**	

Differences from CVV	**9**	**27**	**-**	**-**

Table S3. Amino acid differences between A/Ohio/28/2016 CVV and the 2010.1 clade represented by A/Indiana/27/2018 variant strain. Annotations include mutations within putative epitope and receptor binding sites.

H3 Amino Acid Residue	A/Ohio/28/2016 CVV	A/Indiana/27/2018	3.2010.1 consensus	Annotations
56	H	Y		
138	A	S	S	
142	R	G		
145	K	N	N	Site A
156	H	R		
158	N	D		
189	K	T		Site B, RBS
209	S	N		
Differences from consensus		**-**	**2**	
Differences from CVV		**8**	**-**	

Table S4. Amino acid differences between A/Ohio/28/2016 CVV and A/Iowa/60/2018 HuVac. Annotations include mutations within putative epitope and receptor binding sites.

H3 Amino Acid Residue	A/Ohio/28/2016 CVV	A/Iowa/60/2018 HuVac	Annotations
3	L	I	
9	N	S	
10	M	T	
33	Q	R	
45	S	N	
46	F	S	
48	T	I	
53	N	D	Site C
62	E	G	
82	N	K	
92	K	R	
94	H	Y	
121	N	K	
131	T	K	
133	D	N	Site A, RBS
135	A	T	RBS
140	K	I	
142	R	G	
145	K	S	Site A
159	F	Y	

160	K	T	
165	E	N	
171	N	K	
186	A	G	Site B
193	S	F	Site B, RBS
198	A	S	
199	A	S	
203	I	T	
223	V	I	RBS
238	R	K	
278	N	K	Site C
280	A	E	
312	N	S	
323	I	V	

Table S5. Percentage of macroscopic lung lesions and microscopic lung and trachea scores of pigs.

Clade Group	Pig #	Macroscopic Lung Lesions (%)	Microscopic Lung Lesions Score (0–22)	Microscopic Trachea Lesions Score (0–8)
1990.1	446	0.4	0.0	3.0
	447	0.0	0.0	0.0
	448	0.0	0.0	4.0
1990.4.A	451	0.0	2.0	1.0
	452	0.6	0.5	4.0
	453	0.0	1.0	2.5
1990.4.B.2	456	1.3	3.0	4.0
	457	0.5	0.0	1.0
	458	1.6	0.0	5.0
NC	441	0.0	0.0	0.0
	442	0.0	0.0	0.0
	443	0.0	0.0	0.0

Table S6. Pig HI data at 0, 5 and 14 dpi.

Clade Group	Pig #	0 dpi	5 dpi	14 dpi
	441	<10	<10	-
	442	<10	<10	-
NC	443	<10	<10	-
	444	<10	<10	<10
	445	<10	<10	<10
1990.1	446	<10	10	-
	447	<10	10	-
	448	<10	10	-
	449	<10	NA	160
	450	<10	NA	160
1990.4.A	451	<10	10	-
	452	<10	10	-
	453	<10	20	-
	454	<10	NA	320
	455	<10	NA	320
1990.4.B.2	456	<10	10	-
	457	<10	20	-
	458	<10	10	-

	459	<10	NA	320
	460	<10	NA	320

NA (not applicable)

Dash indicates pigs that were necropsied at 5 dpi.

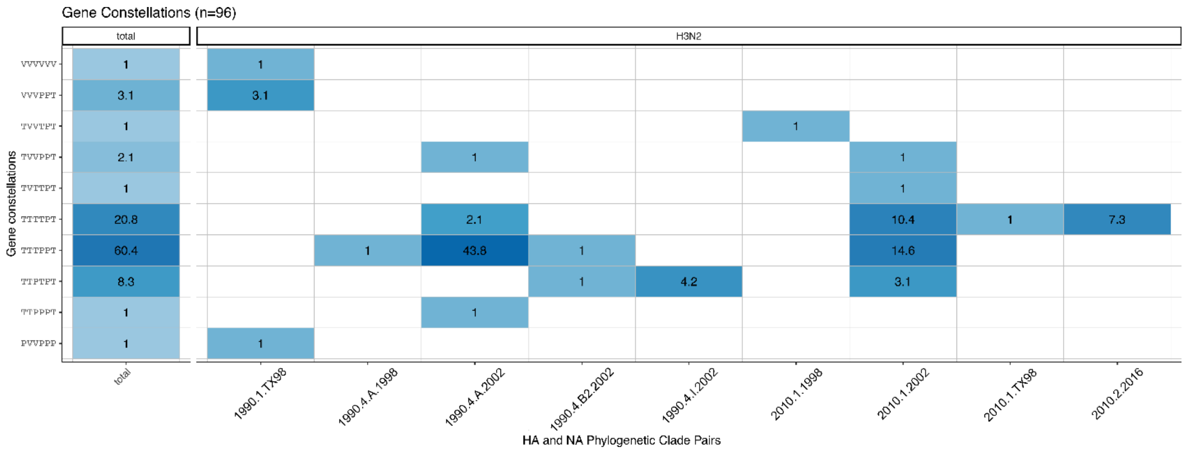

Figure S1. Proportions of detected H3N2 influenza A virus in swine with whole genome data collected in 2020 in the USDA influenza A virus in swine surveillance system. The x-axis reflects the paired genetic clade of hemagglutinin (HA) and neuraminidase (NA), and the y-axis reflects the evolutionary lineage (triple-reassortant, T; H1N1pdm09, P; live attenuated vaccine virus associated, V) of the internal genes in the order of PB2-PB1-PA-NP-M-NS.
